# Tofacitinib treatment aggravates *Staphylococcus aureus* septic arthritis, but attenuates sepsis and enterotoxin induced shock in mice

**DOI:** 10.1038/s41598-020-67928-0

**Published:** 2020-07-02

**Authors:** Anders Jarneborn, Majd Mohammad, Cecilia Engdahl, Zhicheng Hu, Manli Na, Abukar Ali, Tao Jin

**Affiliations:** 1 Department of Rheumatology and Inflammation Research, Institute of Medicine, Sahlgrenska Academy at University of Gothenburg, Guldhedsgatan 10A, 413 46 Göteborg, Sweden; 2 Department of Rheumatology, Sahlgrenska University Hospital, Göteborg, Sweden; 3 Department of Microbiology and Immunology, The Affiliated Hospital of GuiZhou Medical University, Guiyang, China

**Keywords:** Bacterial infection, Rheumatoid arthritis, Septic arthritis, Drug safety, Immunosuppression, Sepsis, Infection

## Abstract

Tofacitinib, a janus kinase inhibitor, is a novel immunosuppressive drug for treatment of rheumatoid arthritis (RA). Septic arthritis (SA) and sepsis caused by *Staphylococcus aureus* (*S. aureus*), for which RA patients are at risk, are infections with high mortality. The aim of this study was to investigate the effect of tofacitinib on *S. aureus* infections using mouse models. In vitro tofacitinib treated mouse splenocytes were stimulated with *S. aureus* derived stimuli. Mice pre-treated with tofacitinib were inoculated intravenously with either arthritogenic- or septic doses of *S. aureus*. Arthritis severity and mortality were compared between groups. Additionally, pre-treated mice were challenged with staphylococcal toxin TSST-1 to induce shock. Tofacitinib inhibited splenocyte proliferation and IFN-γ production in response to TSST-1 and dead *S. aureus*. In SA, tofacitinib treatment aggravated arthritis with more severe bone erosions. However, in sepsis, treated mice displayed significantly prolonged survival compared to controls. Similarly, in staphylococcal enterotoxin-induced shock tofacitinib pre-treatment, but not late treatment dramatically reduced mortality, which was accompanied by decreased levels of TNF-α and IFN-γ. Our findings show that tofacitinib treatment increase susceptibility of SA in mice, but has a positive effect on survival in *S. aureus*-induced sepsis and a strong protective effect in toxin-induced shock.

## Introduction

*Staphylococcus aureus (S. aureus)* is a remarkably resourceful bacterium. Its area of activity ranges from persistent colonization in human nasal flora, approximately 20% of the population^1^, to potentially lethal infections such as endocarditis and septic arthritis (SA). Emergence of highly virulent and antibiotic resistant *S. aureus* strains is a serious challenge worldwide^2^. *S. aureus* is one of the top culprits in the development of sepsis^3^ and *S. aureus* bacteremia has a mortality of around 20%, a number which seems to have stabilized in the past 20 years^4^. SA is another serious infection where *S. aureus* is the most common pathogen (37–56% of the cases), and carries a major risk for loss-of-function of the affected joint^5^. Preexisting joint damage, caused for example by rheumatoid arthritis (RA), is one of the biggest risk factors for septic arthritis^6^, and certain antirheumatic treatments, such as TNF-α-inhibitors, are known to further increase the risk^7^.

Tofacitinib is a new drug for the treatment of various autoimmune diseases. It was first approved for RA^8,9^ in 2014, but is now also used for psoriatic arthritis^10^ and ulcerative colitis^11^, with more diseases awaiting ongoing trials. Tofacitinib belongs to a new class of immunosuppressive drugs, janus kinase (JAK) inhibitors, bridging a gap between conventional and biological disease-modifying antirheumatic drugs (DMARDS)^12^. JAK inhibitors act by inhibiting one or more of the 4 JAKs, in the case of tofacitinib, JAK3 and JAK1, and to a lesser degree JAK2. Inhibition targets the JAK-STAT pathway, which is used by a wide range of cytokines, including IFN-γ, IL-2, IL-4, IL-6^13^. With such a broad range of affected pathways, side-effects during tofacitinib treatment might occur unexpectedly.

It is known that tofacitinib increases the risk for infections, including serious infections, with the risks being comparable to those seen in treatment with TNF-α-inhibitors^14^. However some differences has been found, with herpes zoster standing out as a risk for patients taking tofacitinib compared with other DMARDs^14^. It is still largely unknown how the drug influences infections caused by *S. aureus*, for which patients with RA generally are at higher risk^15^ and have higher mortality^16^. Immunosuppressive treatment in infections can also potentially be beneficial, as it has been shown that corticosteroids or TNF-α-inhibitors in combination with antibiotics alleviate SA in a murine model^17,18^ as well as having a positive effect in some but not all cases of sepsis in humans^19^. Indeed, TNF-α inhibitors have been tested in several randomized controlled trials as anti-sepsis treatment but failed to show convincing positive effect in humans^20,21^, though it might be associated with lower risk of developing sepsis and fatal outcome after serious infection in RA patients^22^.

In the present study we aimed to examine the effect of tofacitinib treatment on SA and sepsis caused by *S. aureus* as well as enterotoxin induced shock in our well-established murine models^23,24^.

## Results

### Tofacitinib inhibits splenocyte proliferation induced by *S. aureus* bacterial components

**Figure 1:**
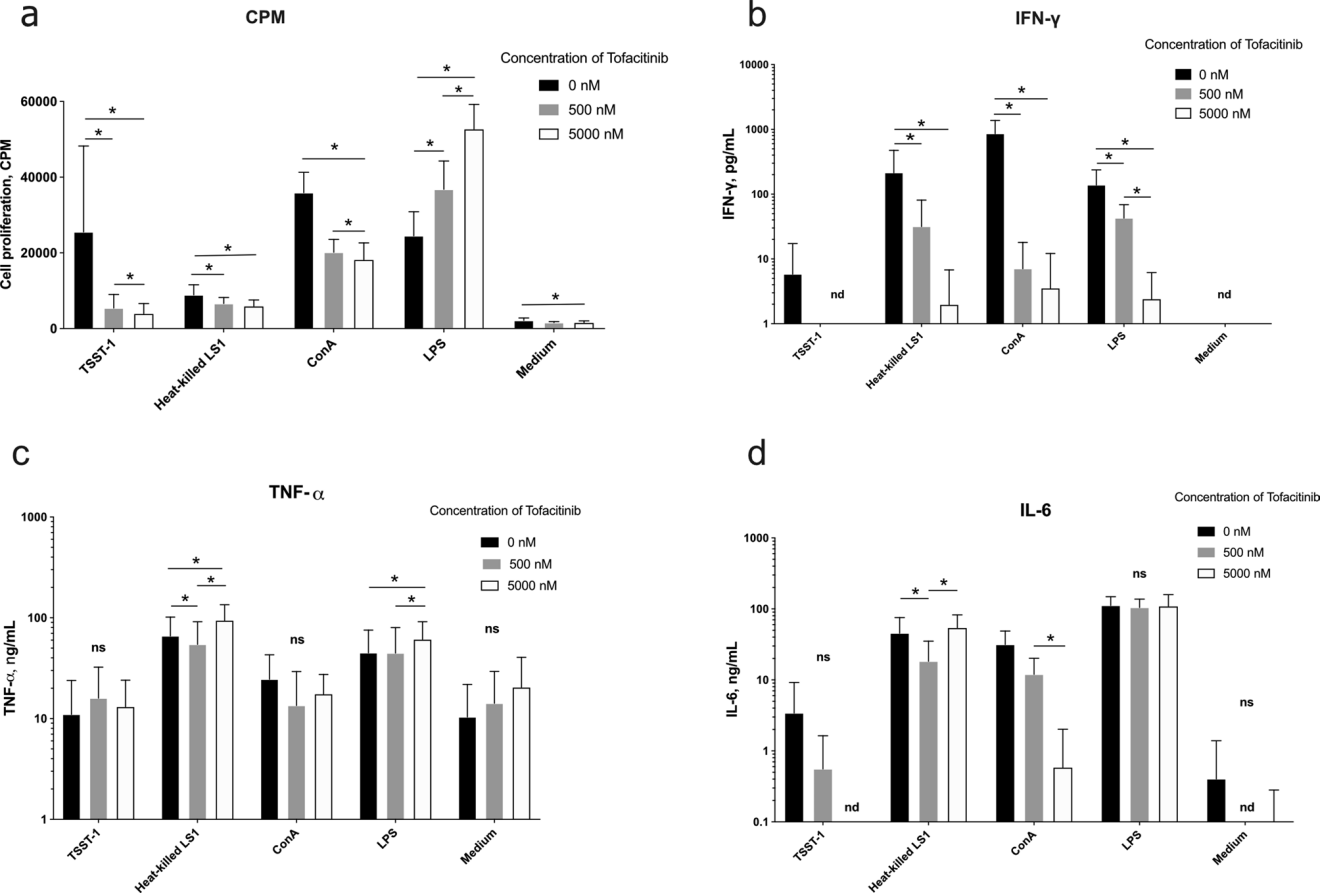
Effect of tofacitinib on mouse splenocytes stimulated with various *S. aureus* components. Mouse splenocytes were isolated from healthy NMRI mice (n = 6). Cells were treated with two different concentrations of Tofacitinib, 5,000 nM or 500 nM, dissolved in DMSO or DMSO only as control, for 2 h. Afterwards, they were stimulated with *S. aureus* toxin TSST-1, heat-killed *S. aureus*, ConA, LPS. or medium only. (**a**) Proliferation of splenocytes, measured as count per minute (CPM). (**b**–**d)** Levels of cytokines IFN-γ (**b**), TNF-α (**c**), IL-6 (**d**) in supernatants from the splenocyte cultures. Values are mean ± SEM. *p* values were determined using the Wilcoxon matched-pairs signed rank test. *p < 0.05; ns, not significant; nd, not detected.

To investigate whether tofacitinib has the potential to impact immune proliferation induced by *S. aureus*, mouse splenocytes pre-treated with tofacitinib were stimulated with *S. aureus* bacterial components including toxic shock syndrome toxin-1 (TSST-1) and heat-killed *S. aureus*. Staphylococcal components and positive controls concanavalin A (ConA) and lipopolysaccharide (LPS) significantly induced splenocyte proliferation (Fig. 1a). Tofacitinib treatment resulted in a significant, dose-dependent decrease in proliferation of splenocytes when stimulated with TSST-1, heat-killed *S. aureus,* and ConA (*p* < 0.05). However, stimulation with LPS, interestingly resulted in a significant, dose-dependent increase in proliferation for cells treated with tofacitinib (*p* < 0.05).

### Effect of tofacitinib on cytokine production from splenocytes stimulated with *S. aureus* components

To further investigate how tofacitinib regulates inflammatory response upon stimulation with *S. aureus* components, cytokine release was measured in the supernatant from the cultures of stimulated splenocytes. The most predominant inhibition exerted by tofacitinib was observed in IFN-γ production. Treatment with tofacitinib resulted in a significant decrease in IFN-γ levels in cells stimulated with heat-killed *S. aureus*, ConA, and LPS (Fig. 1b). In the case of TNF-α and IL-6, the inhibitory effect of tofacitinib was less conclusive for different stimuli. Lower dose of tofacitinib exhibited inhibitory effect on TNF production of cells stimulated with heat-killed *S. aureus*, whereas high dose of tofacitinib seems to boost TNF-α release (Fig. 1c). Also, cells stimulated with LPS showed increased production of TNF-α when treated with the highest concentration of tofacitinib compared with controls (*p* < 0.05) (Fig. 1c). Stimulation with ConA showed a significant decrease in levels of IL-6 from cells treated with the higher dose of tofacitinib compared to the lower dose (*p* < 0.05), and a trend towards decreased levels between lower dose of tofacitinib and dimethyl sulfoxide (DMSO) controls (*p* = 0.06) (Fig. 1d). Our data demonstrate that in vitro tofacitinib treatment regulates immune responses upon stimulation with *S. aureus* bacterial components.

### Tofacitinib treatment aggravates *S. aureus* septic arthritis

**Figure 2:**
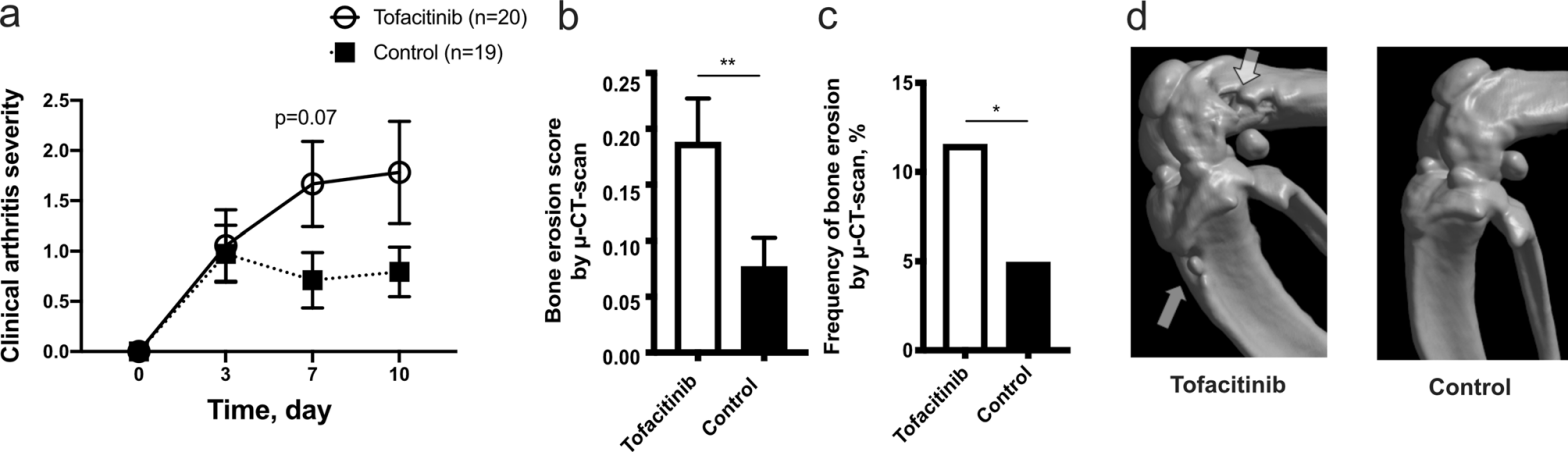
Treatment with tofacitinib aggravates *S. aureus* septic arthritis in mice. NMRI mice were pretreated with tofacitinib (n = 20) or vehicle only as control (n = 19) for 3 days before inoculation and throughout the remainder of the experiment. Mice were inoculated intravenously with arthritogenic dose of *S. aureus* Newman (8.9 × 10^6^–1 × 10^7^ CFU per mouse). (**a**) The severity of clinical arthritis observed for 10 days after inoculation. (**b**–**c**) Bone erosions evaluated with μ-CT. Joints were scanned and reconstructed as a 3D-image. The severity (**b**) and frequency (**c**) of bone erosion compared between groups as seen on day 10 post infection. (**d**) Examples of representative images from μ-CT-scans of a knee with bone erosion and one without erosion. Arrows indicate bone erosion. Values are mean ± SEM. Data were pooled from two independent experiments. *p* values were determined using the Mann–Whitney U test (**a**, **b**) and chi-square test (**c**). *p < 0.05; **p < 0.01; ns, not significant.

To study whether tofacitinib treatment impacts the course of SA caused by *S. aureus* in mice, mice pretreated with the drug or vehicle-only as control were inoculated intravenously (iv) with an arthritogenic dose of *S. aureus* Newman strain. About 40% of the mice presented with arthritis by day 3. By day 7 mice treated with tofacitinib tended to develop more severe arthritis compared to control mice (*p* = 0.07) (Fig. 2a). A total of 4 tofacitinib-treated mice and 2 control mice died during the course of the experiments. All joints were scanned with μ-computer tomography (μ-CT) to examine the degree of bone erosion. The difference between groups was clearer than what was observed clinically, with the tofacitinib treated mice displaying significantly more frequent and severe bone erosions (Fig. 2b, c). Figure 2d demonstrates representative 3D-images of a knee joint with erosions versus an unaffected knee following inoculation with arthritogenic dose of *S. aureus*. Mice lost a total of between 10–20% of their weight during the course of infection. No significant difference was observed between the groups. Kidneys were examined macroscopically for abscess formation and homogenized for colony forming units (CFU) counts, which reflects host ability to clear bacteria. No difference was seen in either abscess formation or CFU counts between groups.

### Mice with septic arthritis treated with tofacitinib show significantly increased levels of IL-6

Sera collected at the end of the first septic arthritis experiment was analyzed for levels of IL-6, TNF-α, IFN-γ, MCP-1 and RANKL by ELISA. There was a significant increase in IL-6 levels in mice treated with tofacitinib compared to controls (*p* = 0.0003). For the other cytokines examined, there was a slight trend in the same direction with higher levels in treated animals, except for RANKL, however none showed significant difference (Table 1).

**Table 1:** Cytokine levels (pg/mL) in serum from mice with staphylococcal septic arthritis.

	IL-6	TNF-α	IFN-γ	MCP-1	RANKL
Tofacitinib	132.9 ± 19.4†	222.5 ± 122.1	12.3 ± 2.1	112.8 ± 35.5	340.5 ± 135.9
Controls	48.9 ± 9.4	159.7 ± 55.8	9.6 ± 1.6	73.5 ± 17.7	379.8 ± 80.71

NMRI mice treated with tofacitinib (n = 8) or vehicle only as control (n = 9) were inoculated with an arthritogenic dose of *S. aureus* Newman (8.9 × 10^6^ CFU/mouse). Mice were sacrificed on day 10 and serum was collected and analyzed for cytokines. All values are pg/mL. Data were shown as mean ± SEM. †*p* = 0.0003 versus control group by Mann–Whitney U test.

### Tofacitinib treatment prolonged survival in mice with sepsis caused by *S. aureus*

**Figure 3:**
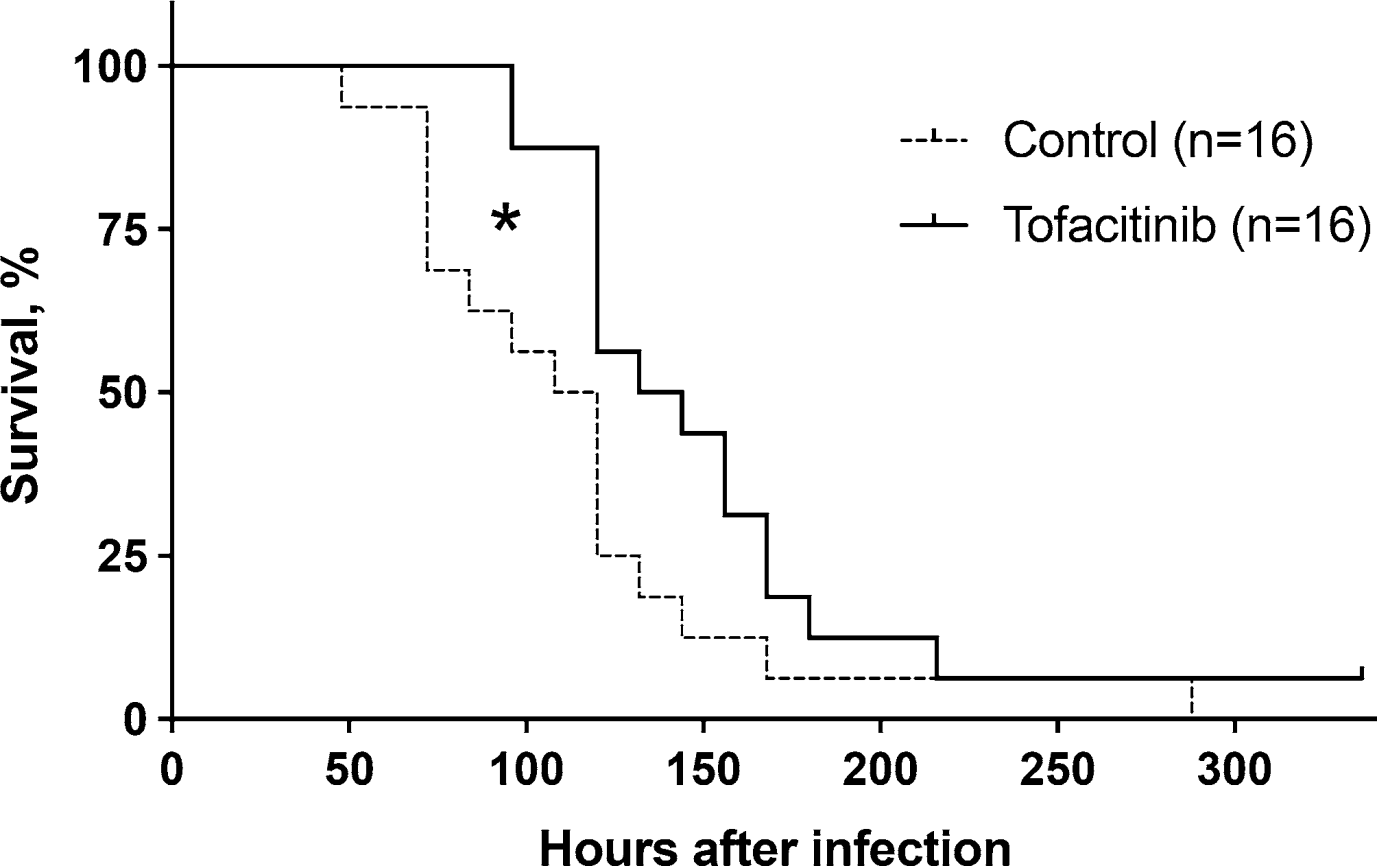
Treatment with tofacitinib prolongs survival of mice with *S. aureus*-induced sepsis. NMRI mice were pretreated with either tofacitinib (n = 16) or vehicle only as control (n = 16) for 3 days before bacterial inoculation and for the remainder of the experiment. On day 0, mice were inoculated with a septic dose of toxin-producing *S. aureus* stain AB-1 (1.8 × 10^7^–6 × 10^7^ CFU per mouse). Data were pooled from two independent experiments. P value was determined using the log-rank (Mantel-cox) test. *p < 0.05.

To study the effect of tofacitinib treatment on staphylococcal sepsis, mice pre-treated with tofacitinib or vehicle only for control were injected iv with a septic dose of *S. aureus* AB-1 strain, which produces the superantigen staphylococcal enterotoxin A (SEA). The first mouse died after 48 h and only 1 mouse survived by the end of the experiment (336 h after infection). Mice treated with tofacitinib survived significantly longer than the control mice (*p* = 0.01) (Fig. 3). Results were pooled from two independent experiments.

### Tofacitinib protected mice against *S. aureus* enterotoxin induced shock

**Figure 4:**
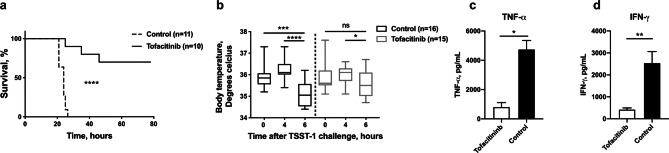
Pre-treatment with tofacitinib protects lethality from *S. aureus* toxic shock. BALB/c mice were treated with tofacitinib or vehicle only as control starting 3 days pre-challenge and throughout the whole experiment. On day 0, mice were injected ip with 10 μg of TSST-1 followed by ip 170 μg of LPS 4 h later. (**a**) Survival of toxin-challenged mice treated with tofacitinib (n = 10) or vehicle only as control (n = 11). Experiment terminated after 78 h. (**b**) Body temperature of tofacitinib treated mice (n = 16) or controls (n = 15) measured via infrared thermometer detection at time of TSST-1 injection, after 4 h at the time of LPS injection and again at 6 h post-TSST-1 injection. (**c**–**d**) Cytokine levels of tumor necrosis factor-α and interferon-γ measured in serum collected at 24 h after TSST-1 injection of mice pretreated with tofacitinib (n = 5) or vehicle only controls (n = 5). Values are mean ± SEM. P values were determined using the log-rank (Mantel-cox) test (**a**) or the Mann–Whitney U test (**b**–**d**). *p < 0.05; **p < 0.01; ***p < 0.001; ****p < 0.0001; ns, not significant.

As *S. aureus* toxins play a vital role in fatal *S. aureus* sepsis, we further evaluated the effect of tofacitinib on enterotoxin induced shock in mice. Mice pre-treated with tofacitinib or vehicle-only were challenged intraperitoneally (ip) with a combination of TSST-1 and LPS. Within 27 h all of the control animals were dead. In sharp contrast to this, the first death of a mouse receiving tofacitinib was 25 h post injection, and 70% survived and recovered by 78 h hours at which time the experiment was terminated. The difference between groups was highly significant (*p* < 0.0001) (Fig. 4a). Body temperature of the mice was measured at set intervals and displayed a significant difference between groups, with control mice showing a significant decline in temperature towards hypothermia as symptoms worsened (*p* = 0.009), while tofacitinib prevented such body temperature changes (Fig. 4b).

### Tofacitinib downregulated TNF-α and INF-γ production in toxic shock mice

It is known that uncontrolled activation of T cells and overwhelming cytokine (importantly TNF-α and IFN-γ) storm upon staphylococcal toxin challenge are the direct cause of fatality in enterotoxin induced shock in mice. Importantly, cytokine analysis from the blood of mice challenged with enterotoxin shock showed significantly lower levels of IFN-γ (*p* = 0.0079) and TNF-α (*p* = 0.0159) in tofacitinib treated group compared to control group (Fig. 4c), strongly suggesting that tofacitinib downregulates the production of proinflammatory cytokines in mice with enterotoxin induced shock. T-cells are central in the pathology of superantigen response and the effects of tofacitinib are known to affect these cells in a number of ways^25^. The T-cell population, total CD3+ as well as CD4+CD8− and CD4−CD8+, was significantly higher in blood of mice treated with tofacitinib compared to controls when challenged with TSST-1 and LPS (Supplementary Fig. S1). Further analysis showed that activated T-cells (CD3+CD69+) was also significantly higher in tofacitinib treated mice (Supplementary Fig. S1).

### Late treatment with tofacitinib does not protect against toxic shock

**Figure 5:**
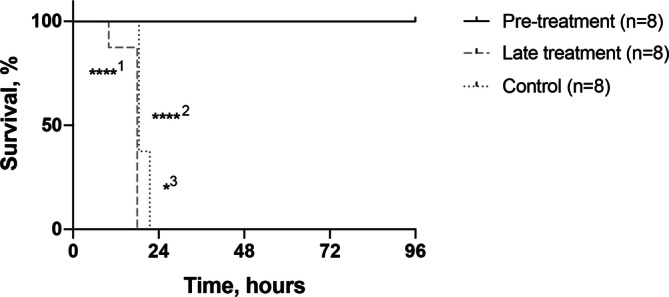
Timing of treatment with tofacitinib is essential for protective effect in toxic shock. BALB/c mice were either injected with tofacitinib subcutaneously (sc) 4 h before and at time of TSST-1 injection (pre-treatment, n = 8), or 6 and 10 h after TSST-1 injection (late treatment, n = 8). A control group was sc given vehicle of PBS and DMSO only (controls, n = 8). Survival of toxin challenged mice was registered until 96 hrs after TSST-1 challenge. *p* values were determined using the log-rank (Mantel-cox) test. ^1^Pre-treatment versus late treatment; ^2^Pre-treatment versus control; ^3^Late treatment versus control; *p < 0.05; ****p < 0.0001.

To further examine the importance of timing of the effect of tofacitinib treatment on toxic shock, mice were pre-treated with two doses of the drug subcutaneously (sc) before toxin challenge or treated after toxin injection when mice had begun to show symptoms of shock. As seen in previous experiments, pre-treatment significantly improved survival and rescued all animals from death (Fig. 5). However, the late treatment aggravated the disease, with those mice dying slightly earlier than the control group receiving only vehicle.

## Discussion

Our present study demonstrates dual effects of tofacitinib treatment in *S. aureus* systemic infections, with potential risks and benefits depending on the type and location of infection.

In the current study, tofacitinib displayed a robust downregulation on IFN-γ production by splenocytes upon stimulation with *S. aureus* components in vitro*.* IFN-γ is known to be protective in septic arthritis, as IFN-γ receptor deficient mice developed more severe and frequent septic arthritis than wildtype mice^26^. In addition, NK-cells that are protective in *S. aureus* septic arthritis^27^ are known to be downregulated by tofacitinib treatment^14^. We speculate that at the onset of disease immune responses for bacteria elimination including IFN-γ production was blocked by tofacitinib pretreatment and therefore mice were more vulnerable to septic arthritis. Of note, the IFN-γ levels measured in the septic arthritis mice did not show any significant difference on day 10 post infection. This might be due to the reason that the IFN-γ levels are generally very low at the later stage of infection even without active treatment and any difference at this timepoint might not be caught.

Surprisingly, when mice were inoculated with a septic dose of *S. aureus* strain AB-1 that produces a large amount of staphylococcal enterotoxin, the tofacitinib treatment had a beneficial effect, with treated mice surviving significantly longer. Actually, it has been shown that a JAK2 inhibitor increase survival in mice with gram negative bacterial sepsis induced by cecal ligation puncture by downregulating NF-κB activation, macrophage activation, and proinflammatory cytokine release^28^. Also, gene delivery of suppressor of cytokine signaling (SOCS) proteins that mediate a negative feedback on JAK/STAT protect against lethal endotoxic shock, which was accompanied by decreased serum levels of TNF-α^29^. Furthermore, Tarkowski et al. previously demonstrated that the mice deficient in the receptor for IFN-γ had better survival in *S. aureus* sepsis^26^, which supports our findings that tofacitinib prevents *S. aureus* sepsis at least in the mouse model of acute *S. aureus* sepsis.

In line with the results obtained from sepsis model, in the model of enterotoxin induced shock tofacitinib treatment strongly impacted the host response to the challenge of toxins and remarkably reduced the mortality rate. It is known that decreased body temperature correlate to and are a hallmark of severity of the disease in mice with enterotoxin shock^30^. Indeed, less fluctuation of body temperature in mice given tofacitinib treatment after toxin challenge supports the mortality data. TNF-α and IFN-γ are known to be the key cytokines in enterotoxin induced shock model, as both treatment with neutralizing antibodies and gene deficiency protect the lethality^30–32^. In current study, tofacitinib treatment robustly downregulated the release of both TNF-α and IFN-γ in mice with enterotoxin induced shock, which explains the milder shock symptoms and attenuated mortality rate in tofacitinib treated mice.

In the tofacitinib treated mice we found higher numbers of both CD4+ and CD8+ T-cells. While T-cells are initially massively activated in shock, their number however decreases during the course of disease and lymphopenia is common^33,34^. It has been shown that lymphopenia is common in sepsis and could be a marker for increased mortality^35^. While T-cell numbers were higher in tofacitinib treated animals, the levels of IFN-γ and TNF-α were both significantly lower than in control animals. This might indicate that while T-cell numbers were high, tofacitinib may have inhibited their final differentiation and ability to contribute to the cytokine storm responsible for the toxic shock. It has been shown that the JAK-STAT-pathway is involved in differentiation of Th-cells including those affected by IFN-γ, IL-4, IL-6 and IL-23^13,25,36^.

It is well accepted that in sepsis a hyperinflammatory process occurs at the first days of infection and at the same time a state of immunosuppression starts to set in. These processes seem simultaneous with strong leaning to proinflammation in the beginning followed by a more prolonged period of suppression^37^. With this in mind an ideal therapy against sepsis would aim to balance immune response towards effective elimination bacteria while maintaining a minimal degree of collateral organ damage. Some immunosuppressive agents have been tried in infectious disease, mostly in sepsis, without satisfactory results, including corticosteroids and TNF-α inhibitors, which was efficient in mouse models^19–21^. The failure might be due to the inherent issues with most of the studies since the sepsis conditions in clinical trials were very heterogenous in causative pathogens that might lead to differences in immunological response as well as different stages of sepsis when the treatments were introduced. Apparently, just as TNF-α inhibitors, tofacitinib treatment is also able to modulate hyperinflammation at the early phase of disease, reduce the acute organ damage, and rescue mice from *S. aureus* sepsis and enterotoxin shock. However, it might lead to unwanted deterioration if the disease has progressed and immune paralysis has already established when tofacitinib treatment is planned. Indeed, our results demonstrated that treatment with tofacitinib after debut of shock symptom resulted in an opposite effect, with treated mice displaying a faster death than control animals.

What is the clinical implication of our findings? Our data suggest that patients on tofacitinib treatment may have a higher risk of developing more-severe and frequent bone destructions of septic arthritis. More caution should be taken for patients who are undergoing tofacitinib treatment and have unexplained acute synovitis. At the same time, those patients receiving tofacitinib might have a lower risk to develop fatal sepsis when they encounter a severe infection. However, future clinical and population studies are warranted to ascertain our conclusion from the experimental settings.

## Methods

### Mice and animal experiment ethics statement

Female NMRI mice and female BALB/c mice, 6–12 weeks old, purchased from Charles River Laboratories, Sulzfeld, Germany, were housed in the animal facility of the Department of Rheumatology and Inflammation Research, University of Gothenburg. Mice were kept under standard environmental conditions of temperature and light and were fed laboratory chow and water ad libitum. Experiments were approved by the Animal Research Ethical Committee of Gothenburg, and animal experimentation guidelines were strictly followed.

### In vitro spleen cell stimulation

Preparation and stimulation of splenocytes from healthy NMRI mice was performed as previously described^38^. The cells were treated with different concentrations of tofacitinib dissolved in DMSO (500 nM and 5,000 nM) or DMSO alone as control for 2 h. Cells were then stimulated with *S. aureus* components including TSST-1 (100 ng/mL), heat-killed *Staphylococcus aureus* LS-1. LPS (1 μg/mL) and ConA (1.25 μg/mL) served as positive controls, whereas medium served as negative control. Two independent experiments were repeated with similar outcomes and results were pooled.

### Treatment with tofacitinib

For in vivo pre-treatment studies mice were administered tofacitinib (Hölzel Diagnostika Handels GmbH, Germany) via an Alzet mini-osmotic pump (model 2002, Durect Corporation). Content was prepared for a drug delivery of 15 mg/kg/day for all experiments. Tofacitinib was dissolved in DMSO and delivered with a vehicle solution containing 10% PEG-300, 40% water and tofacitinib dissolved in DMSO or only DMSO for controls. Pumps were surgically implanted subcutaneously on the back of each mouse, per manufacturer’s instructions. For experiment comparing pre- and late treatment mice were treated by a 0.2 mL sc injection of a dose of 50 mg/kg tofacitinib dissolved in 25% DMSO and 75% PBS. For control mice were injected with the same volume of 25% DMSO and 75% PBS only.

### Protocol for *Staphylococcus aureus* septic arthritis

Two in vivo experiments for septic arthritis were performed independently. NMRI mice were randomized for implantation with osmotic mini-pumps containing tofacitinib (n = 20) or vehicle only as controls (n = 19). Three days after pump implantation mice were inoculated iv via tail vein with an arthritogenic dose of *S. aureus* Newman strain (8.9 × 10^6^–1 × 10^7^ CFU/mouse). Clinical signs of arthritis were monitored as previously described^39^. Briefly, blinded observers (T.J. M.M.) scored each paw for signs of arthritis on a scale of 0–3 and an index was constructed by adding the scores from all 4 limbs for each mouse, whereby each mouse could be awarded 12 points as maximum. On day 10 after infection mice were sacrificed by cervical dislocation after anesthesia with ketamine hydrochloride (Pfizer AB, Sweden) and medetomidine (Orion Pharma, Finland). Kidneys and blood were collected, and all four limbs were removed and put in formalin for radiographic examination by μ-CT for bone erosions. Whole blood centrifuged for 15 min at 13,200 rpm after clot formation. Serum was removed and stored at − 20 °C until analysis. For analysis of cytokines or chemokines, enzyme-linked immunosorbent assays (ELISA) kits were used for IL-6, TNF-α, IFN-γ, MCP-1, RANKL DuoSet ELISA (R&D systems, Europe, Ltd.), according to the manufacturer’s directions. Kidneys from mice in arthritis experiments were visually examined and blindly judged by two observers (M.M. and M.N.) for degree of abscess formation. A scoring system of 0–3 was used as previously described^40^. Kidneys were then homogenized, serially diluted with PBS and spread on agar plates containing 5% horse blood. Plates were incubated at 37 °C for 24 h and bacteria were quantified as CFUs. The experiments had similar outcomes and results were pooled.

### Micro-computed tomography (μ-CT)

Joints were collected at the end of arthritis experiments and examined for degree of bone erosions by radiographic imaging with μ-CT as previously described^41^. 3D-reconstructions of joints were blindly evaluated (A.J. and Z.H. and T.J.) for extent of bone destruction and graded from 0–3 (0, healthy joint; 1, mild bone destruction; 2, moderate bone destruction; 3, marked bone destruction). Severity of erosion was analyzed by grouping the scores of all individual joints in each treatment group respectively and comparing the scores. Frequency of erosion was described as percentage of positive joints out of total joints in each group. A score of ≥ 1 was considered positive for erosion.

### Protocol for *Staphylococcus aureus* sepsis

To study the effect of tofacitinib on sepsis, two separate experiments were performed. In each experiment NMRI mice were implanted with mini-osmotic pumps as described above with 8 mice in each group per experiment. Three days after implantation the mice were injected through the tail vein with a septic dose of 1.8 × 10^7^–6 × 10^7^ CFU of the SEA-producing *S. aureus* strain AB-1, which produces superantigen staphylococcal enterotoxin A (SEA). Survival of the mice was checked at least every 12 h and if a mouse was deemed too ill to survive until the next checkpoint it was sacrificed and considered dead due to sepsis. Severity of illness was based on fur quality, decreased movement, signs of pain and discomfort.

### Protocol for toxin induced shock

BALB/c mice were pre-treated via mini-osmotic pump with either tofacitinib or vehicle as described above. On day 3 they were injected ip with 10 μg of the *S. aureus* superantigen TSST-1 followed by another ip injection after 4 h of 170 μg LPS. Neither of these doses is lethal in itself, but together they induce toxic shock. Body temperature was measured by an infrared thermometer (Braun Thermoscan IRT6020) on the skin at 0, 4 and 6 h after TSST-1 injection. Mortality and signs of discomfort was evaluated at regular intervals, at least every 8 h. If a mouse was deemed too ill to survive until the next timepoint it was sacrificed and considered dead due to shock. Mice were observed until 78 h after injection of TSST-1.

For fluorescence-activated cell sorting (FACS)-analyses and cytokine measurements, mice were challenged with toxins as described above in separate experiments. At around 16–18 h after injection of TSST-1 these mice were anesthetized, and blood was collected.

For comparison between pre- and late treatment with tofacitinib in the shock model, BALB/c mice were divided into three groups with 8 mice in each. One group received tofacitinib by sc injection 4 h before and at the time of TSST-1 challenge. The second group received tofacitinib 6 h and 10 h after TSST-1 challenge. The third group received only vehicle consisting of DMSO and PBS. At each tofacitinib injection, the groups not receiving active treatment were given an injection of only vehicle.

### FACS-analysis of lymphocyte populations

Blood were collected in heparin filled tubes. Cell suspensions were obtained and stained for surface markers after erythrolysis and fixation. Analysis was performed using FACSVerse (BD) and analyzed with FlowJo version 10 (Tree star). Alive cells were gated on singlet cells and on the alive cells; T cells (CD3+CD19−); activated T cells (CD3+CD19−CD69+); T helper cells (CD4+CD8−); activated T helper cells (CD4+CD8−CD69+); cytotoxic T cells (CD4−CD8+); activated cytotoxic T cells (CD4−CD8+CD69+). The following fluorochrome anti-mouse antibodies were used; Peridinin Chlorophyll Protein complex (PerCP)-conjugated anti-CD19 (Biolegend), PE-cyanine 7 (PE-Cy7)-conjugated anti-CD8 (Biolegend), allophycocyanin (APC)-conjugated anti-CD4 (Biolegend), APC-Cy7-conjugated anti-CD3 (BD).

### Statistical analysis

For statistical analysis, GraphPad Prism version 7 and 8 (GraphPad software) were used. To compare the differences between the groups, the Mann–Whitney U test, Wilcoxon test, the log-rank (Mantel-cox) test and Chi-Square test were used and the results are reported as mean ± standard error of the mean (SEM) unless indicated otherwise. The *p* < 0.05 was considered statistically significant.

## Supplementary information

Supplementary file1 (DOCX 494 kb)
