# Supplementary material for: Tofacitinib treatment aggravates Staphylococcus aureus septic arthritis, but attenuates sepsis and enterotoxin induced shock in mice
Source: Sci Rep. 2020 Jul 2;10:10891. doi: 10.1038/s41598-020-67928-0 (PMC7331611; doi:10.1038/s41598-020-67928-0)
Supplement: Supplementary file 1 — Supplementary file1 (DOCX 494 kb) [file 41598_2020_67928_MOESM1_ESM.docx]

**Supplementary Information**

**Tofacitinib treatment aggravates *Staphylococcus aureus* septic arthritis, but attenuates sepsis and enterotoxin induced shock in mice**

Anders Jarneborn^1,2*^, MD; Majd Mohammad^1^, MSc; Cecilia Engdahl^1^, PhD; Zhicheng Hu^1,3^, MD; Manli Na^1^, PhD; Abukar Ali^1^, PhD; Tao Jin^1,2^, MD, PhD.

1. Department of Rheumatology and Inflammation Research, Institute of Medicine, Sahlgrenska Academy at University of Gothenburg, Göteborg, Sweden

2. Department of Rheumatology, Sahlgrenska University Hospital, Gothenburg, Sweden

3. Department of Microbiology and Immunology, The Affiliated Hospital of GuiZhou Medical University, Guiyang, China

***Address of corresponding author:**

Anders Jarneborn, MD

Department of Rheumatology and Inflammation Research,

Institute of Medicine

The Sahlgrenska Academy at the University of Gothenburg

Guldhedsgatan 10A, S-413 46 Göteborg, Sweden

Phone: 46-31-3426475

Fax: 46-31-823925

E-mail: [anders.jarneborn@gu.se](mailto:anders.jarneborn@gu.se)


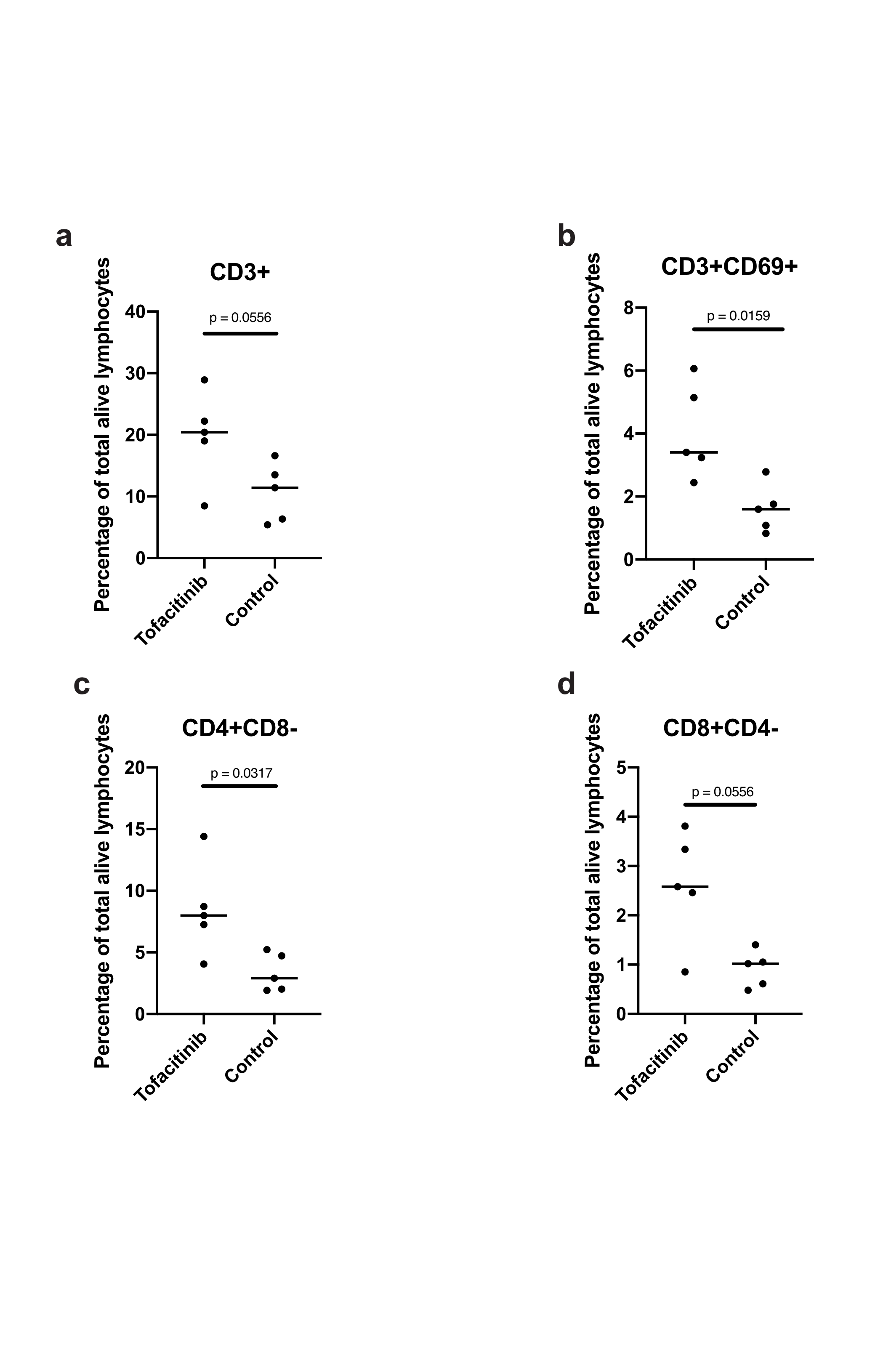


**Supplementary figure S1. Tofacitinib treatment results in higher levels of T-cells in toxin induced shock mice.** T-cell populations in blood were analyzed by FACS in mice pretreated with tofacitinib or vehicle only and challenged with *S. aureus* enterotoxin TSST-1 and LPS. Frequency of T-cells (% CD3+ of alive blood cells) **(a)**, frequency of activated T-cells (% CD3+CD69+) (**b**), frequency of Th-cells (% CD4+CD8-) (**c**), frequency of cytotoxic T-cells (% CD8+CD4-) (**d**). P values were determined using the Mann-Whitney U test.
